# Sucrose Transporter StSUT2 Affects Potato Plants Growth, Flowering Time, and Tuber Yield

**DOI:** 10.3390/cimb45030172

**Published:** 2023-03-22

**Authors:** Hui-Ling Gong, Jin-Bao Liu, Clement Igiraneza, Leonce Dusengemungu

**Affiliations:** 1 School of Life Sciences and Engineering, Lanzhou University of Technology, 287 Langongping Road, Lanzhou 730050, China; 2 Guangdong Hybribio Biotech Co., Ltd., Building 2, National Biomedical Industry Base, Yuzhong Park, Lanzhou 730100, China

**Keywords:** sucrose transporter, StSUT2, potato growth and development, tuber yield

## Abstract

Background: Sucrose transporters (SUTs) mediate sucrose phloem loading in source tissue and sucrose unloading into sink tissue in potatoes and higher plants, thus playing a crucial role in plant growth and development. In potatoes, the physiological function of the sucrose transporters StSUT1 and StSUT4 has been clarified, whereas the physiological role of StSUT2 is not yet fully understood. Methods and Results: This study analyzed the relative expression of StSUT2 compared to that of StSUT1 and StSUT4 in different tissues from potatoes and its impact on different physiological characteristics by using StSUT2-RNA interference lines. Here, we report a negative effect of StSUT2-RNA interference on plant height, fresh weight, internodes number, leaf area, flowering time, and tuber yield. However, our data indicate that StSUT2 is not involved in carbohydrate accumulation in potato leaves and tubers. In addition, the data of the RNA-seq between the StSUT2-RNA interference line and WT showed that 152 genes were differentially expressed, of which 128 genes were upregulated and 24 genes were downregulated, and the GO and KEGG analyses revealed that differentially expressed genes were mainly related to cell wall composition metabolism. Conclusions: Thus, StSUT2 functions in potato plant growth, flowering time, and tuber yield without affecting carbohydrate accumulation in the leaves and tubers but may be involved in cell wall composition metabolism.

## 1. Introduction

Sucrose levels, light, temperature, and gibberellic acid have been identified as the most significant factors that regulate tuber formation and plant development in potatoes (*Solanum tuberosum* L.) [1]. The sucrose supply, the main carbohydrate transported from leaves to sink organs, is essential for growth in most higher plants [2]. In potatoes, as in most plants, soluble sugars are identified not only as playing a role as energy sources and skeleton molecules for plant growth and development but also function as signal molecules to regulate physiological activities and stress responses [3,4].

Sucrose transporters known as SUTs are important proteins for sucrose phloem loading in source tissues and unloading into sink tissues [4,5] Many studies have been undertaken to isolate and characterize different types of SUTs [5,6]; recently, five independent clades have been classified into all plants: SUT1, SUT2, and SUT4 in dicots and SUT2, SUT3, SUT4, and SUT5 in monocots [7]. A phylogenetic study showed that different members of each SUT clade show different functions. In dicots, SUT1 is high-affinity low-capacity, and SUT2 and SUT4 are low-affinity high-capacity [8]. SUT1 was shown as represented by numerous members and participates in different physiological functions of phloem loading and long-distance sucrose transport [3,9]. In contrast, one member represents the SUT2 and SUT4 families and is known to express them at a low level [6]. Further, SUT3, and SUT5 are monocot-specific transporters [8]. Researchers have indicated that SUT4 within different plant species has participated in diverse biological processes [10,11]. StSUT4 in potatoes affects the flowering, tuberization, and shade avoidance response [6], and tonoplast PtaSUT4 in *Populus* plays an important role in biomass and carbon partitioning [12]. Yang et al. also demonstrated that SUT4 plays an important role in regulating fruit growth and ‘plant’ development in the biofuel plant *Jatropha curcas* [13]. On the other hand, sucrose transporter gene PbSUT2 from *Pyrus bretschneideri* enhances the sucrose content in *Solanum Lycopersicum* fruit and leads to early flowering [14]. SUT2 is mostly expressed in sink organs, such as sink leaves, stems, and fruits, in contrast to SUT1, which is primarily expressed in sucrose-exporting source leaves in solanaceous species [15]. Using a BLAST search, it was realized that the majority of expressed sequence tags (ESTs) for SUT2 sucrose transporter-like proteins were from developing young plant organs: The finding of ESTs from SUT2 genes in young, developing tissues, flowers, flower buds, developing roots, and hormone- or insect-damaged tissue materials suggests that SUT2 and SUT1 have distinct roles from one another. In tomatoes, SUT2, which was mostly expressed in mature flower anthers, appears pivotal for pollen loading, tube growth, and phloem unloading [15]. However, SUT2 localization and function in sink tissues of potato plants have not yet been studied to establish its physiological role.

Potatoes (*Solanum tuberosum* L.) play an important role in food security and are among the four largest produced food with maize, rice, and wheat [16]. Previous studies have shown the high necessity of sucrose to induce tuberization in potatoes [17,18]. Tuber filling and potato production yield depend on the efficient transport of sucrose from the leaves to seeds (sinks) [19]. The knockout and overexpression of SUT genes provide genetic evidence that sucrose transporters have essential roles in sucrose transport and plant development [14]. Kühn found the antisense inhibition of StSUT1 in potato tubers impairs early tuber development. They proposed that StSUT1 is either directly involved in phloem unloading in potato tubers or indirectly by regulating the apoplasmic osmolarity via its retrieval function [20]. Chincinska et al. obtained a high tuber production and earlier flowering phenotypes for StSUT4-RNA interference plants compared to wild-type plants and concluded that StSUT4 plays a key role in the interaction between carbon availability and flower-inducing processes, correlating the effects of light quality and quantity on flowering and tuberization [6]. Their study further indicated that starch accumulation, tuber development, and tuberization induction are all affected by StSUT4-RNAi [6]. It was also reported that StSUT1 antisense in potato tubers could probably affect the expression of StSUT2 or StSUT4 [6,21,22]. StSUT2 mRNA and proteins are found in the phloem of sieve elements and are colocalized with StSUT1 and StSUT4 [6]. However, the function of StSUT2 in the growth and development of potatoes still needs further elucidation. Therefore, this study investigated the functional characterization of StSUT2 in the growth, development, flowering, and tuber yield using StSUT2-RNA interference potato plants.

## 2. Materials and Methods

### 2.1. Plant Materials and Growth Conditions

**Figure 1:**
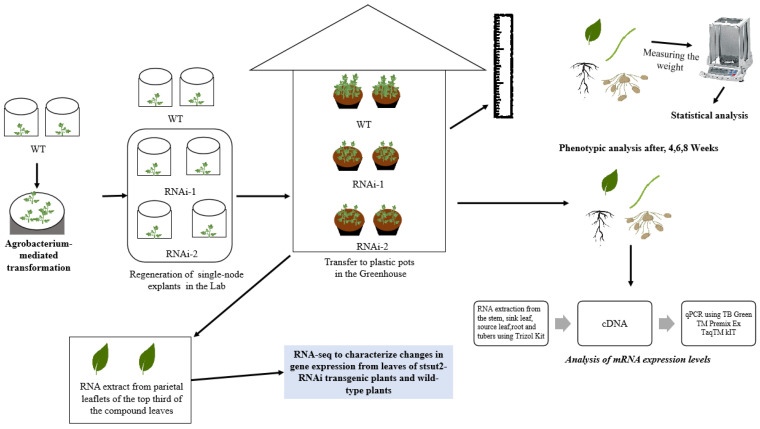
An illustration of the summarized experimental procedure used to analyze potato RNAi-plants.

The potato (*Solanum tuberosum* L.) ‘Shepody’ cultivar and its StSUT2-RNAi transgenic plants (RNAi-1 and RNAi-2 lines) grown in our laboratory in 2019 were used throughout this study. The construction of RNAi transgenic plantlets was completed using the previous study of our lab [23]. Freshly single-node explants were cultured in standard tissue culture media Murashige and Skoog to obtain completely regenerated plantlets before further experiments. Four weeks after the subculture, in vitro plantlets were transferred to plastic pots (13 cm in diameter and 15 cm in depth) containing vermiculite (with four plants per pot). Plants were maintained in greenhouse conditions with a 16 h photoperiod (400 μmol photons m^−2^s^−1^) following a 24/20 °C light/dark cycle with 70% ± 5% relative humidity. Seedlings were watered daily and fertilized once a week with 25% Hoagland nutrient solution [24]. The phenotypic analysis was conducted after plants were cultured for 4, 6, and 8 weeks. In addition, the 4-week-old plantlet parietal leaflets of the top third of the compound leaves were excised and were immediately frozen in liquid N2 and stored at −80 ℃ for RNA extract (Figure 1).

### 2.2. Determination of Phenotypic Analysis of StSUT2-RNAi Plants

The fresh weight aboveground (leaf and stem), and the fresh weight belowground (roots) in each period were measured with a balance. A ruler was used to measure the plant height, and the number of internodes was counted at each experiment period. ImageJ software (Version 1.52s) analyzed the third, fourth, and fifth penultimate leaf areas.

### 2.3. Analysis of Potato Yield

Potato plants were cultured in the greenhouse, and tubers were harvested after 80 days. The yield of tubers per plant and the number of tubers per plant were analyzed statistically.

### 2.4. RNA-Seq

To characterize changes in gene expression from leaves of StSUT2-RNAi transgenic plants and wild-type plants, three replicates of the RNAi-2 line were selected. Beijing Biomic Biotechnology Co. carried out the construction and sequencing of the library, Ltd. Prior to mapping reads to the reference database, we filtered all sequences to remove low-quality reads. Detailed statistical analysis was conducted to describe the gene sequenced, which provided the general information for further transcriptome analysis.

### 2.5. Analysis of mRNA Expression Levels

RNA from the stem, sink leaf, source leaf, root, and tubers were extracted using the uniq-10 strain Trizol kit and then reversed-transcribed into cDNA for qPCR validation using TB Green TM Premix Ex Taq^TM^ kit. Good-quality RNA was used for qPCR, according to Mason et al. [25]. The 2^−∆∆CT^ method was used to calculate the relative expression levels. All sequences of primers and corresponding targets are shown in Table S1.

### 2.6. Statistical Analysis

The SPSS version 17.0 (SPSS Inc., Chicago, IL, USA) and origin programs were used for all statistical analyses. Duncan’s multiple range tests were used to detect significant differences between means at an overall significance level of *p* < 0.05.

## 3. Results

### 3.1. Gene Expression of StSUT1, StSUT2, and StSUT4 in Different Tissues

**Figure 2:**
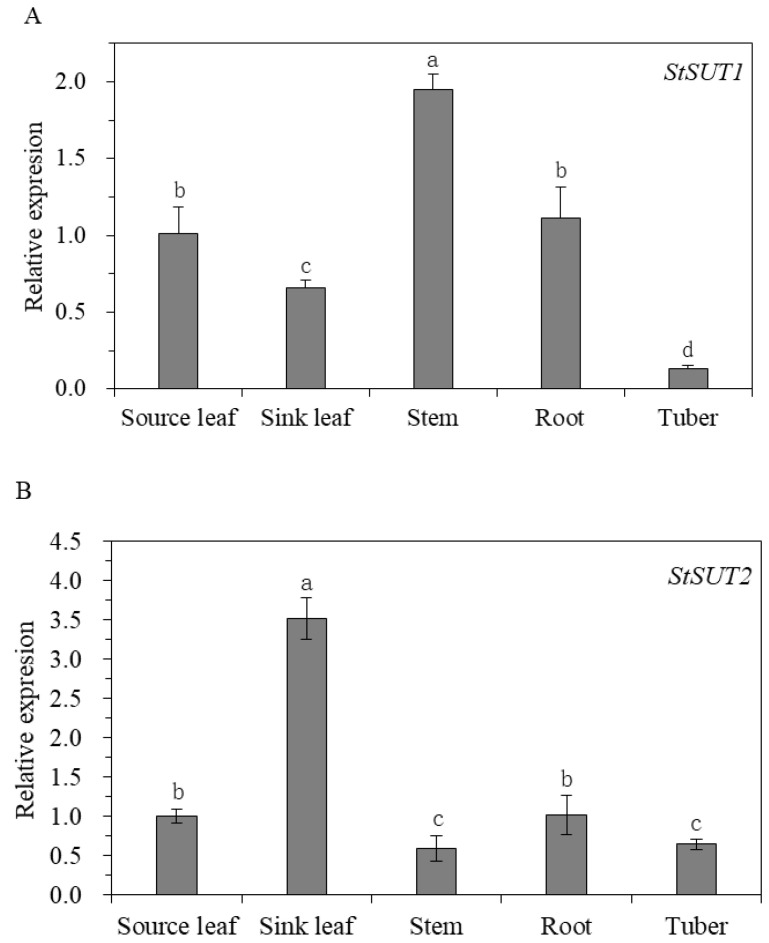

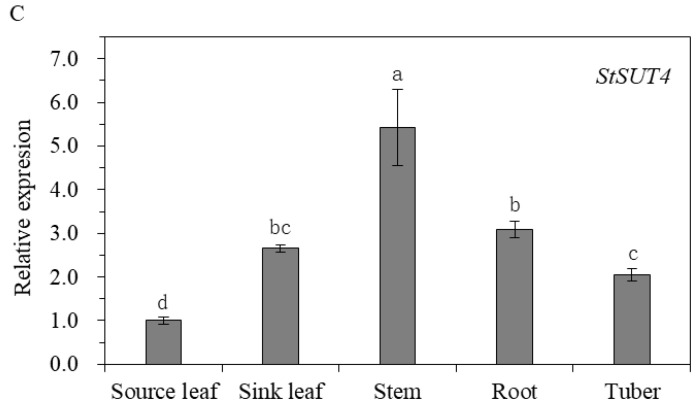
Relative expression of StSUT1 (**A**), StSUT2 (**B**), and StSUT4 (**C**) in different tissues of potato plants by qPCR. According to Duncan’s multiple range test, the same letters are not significantly different at *p* ≤ 0.05.

In potatoes, StSUT1, StSUT2, and StSUT4 were isolated [26]. To determine the gene expression of StSUT1, StSUT2, and StSUT4 in different tissues, qPCR has been performed, and the results indicated that the expression varies among different tissues. The average relative expression pattern of StSUT1 and StSUT4 was 1.94 and 5.41, respectively, in the stem, two- to three-fold higher in comparison with other tissues. StSUT2 expression was 3.52 in sink leaf tissues, remarkably higher than any other potato tissue. StSUT1 and StSUT4 both exhibited the highest expression in the stem, whereas the transcript of StSUT2 was highly abundant in the sink leaf (Figure 2). The StSUT2 level of expression in the roots and source leaf do not differ significantly, with the lowest transcripts in the stem and tuber, as indicated by the results. In contrast, the lowest expressions of StSUT1 and StSUT4 were found in the tuber and source leaf, respectively. StSUT1 shows more expression in the roots and source leaf than in the sink leaf, while the high expression level of StSUT4 was more found in the roots and sink leaf than in the tuber.

### 3.2. Phenotypic Analysis of StSUT2-RNA Interference Plants

#### 3.2.1. Effect of StSUT2-RNA Interference on Potato Vegetative Growth

**Figure 3:**
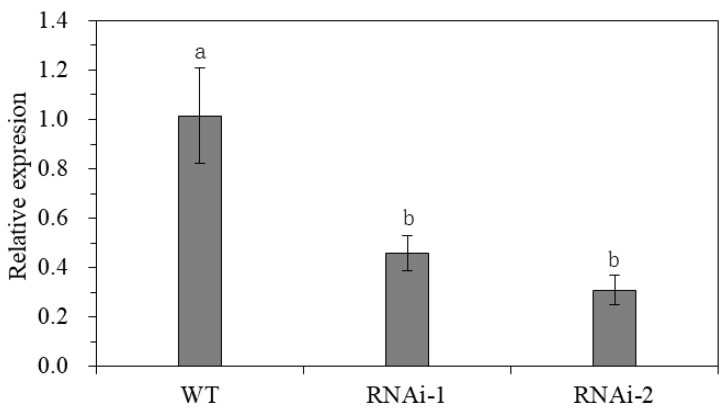
StSUT2 expression level in the leaf of StSUT2-RNA interference lines. According to Duncan’s multiple range test, the same letters are not significantly different at *p* ≤ 0.05.

A gene expression analysis by qPCR revealed the reduction of StSUT2 expression in the StSUT2-RNA interference lines of the RNAi-1 and RNAi-2 plants compared with the wild-type plants (Figure 3). The expression of StSUT2 in the leaf tissues of RNAi-1 and RNAi-2 lines was significantly lower compared to the wild-type, with average relative expressions of 0.45 and 0.31 at 8 weeks, respectively, indicating a two-fold reduction.

**Figure 4:**
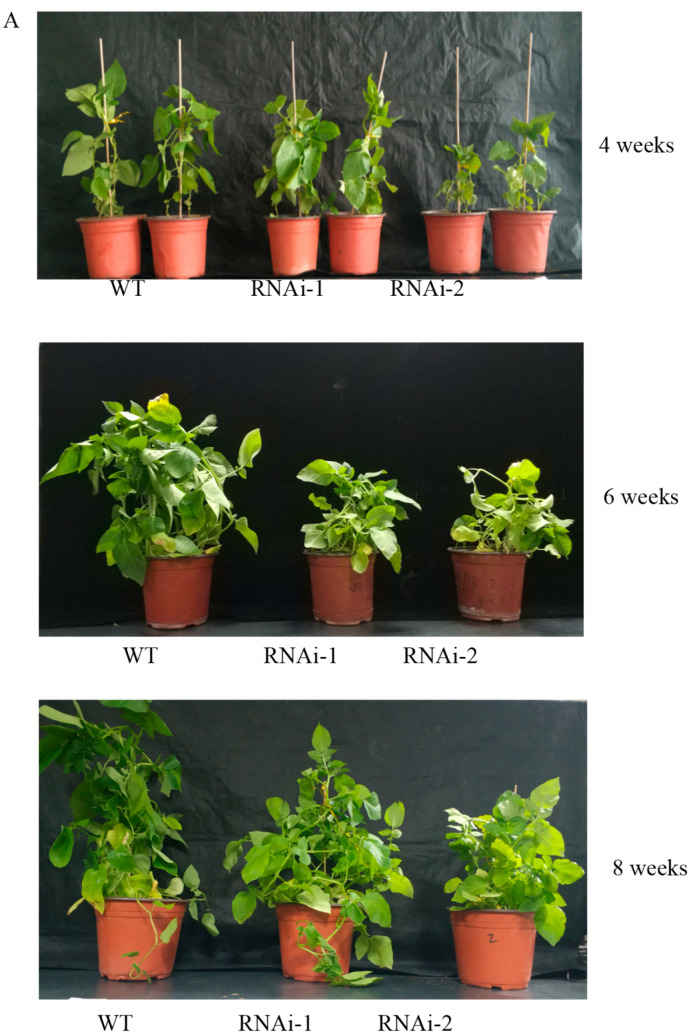

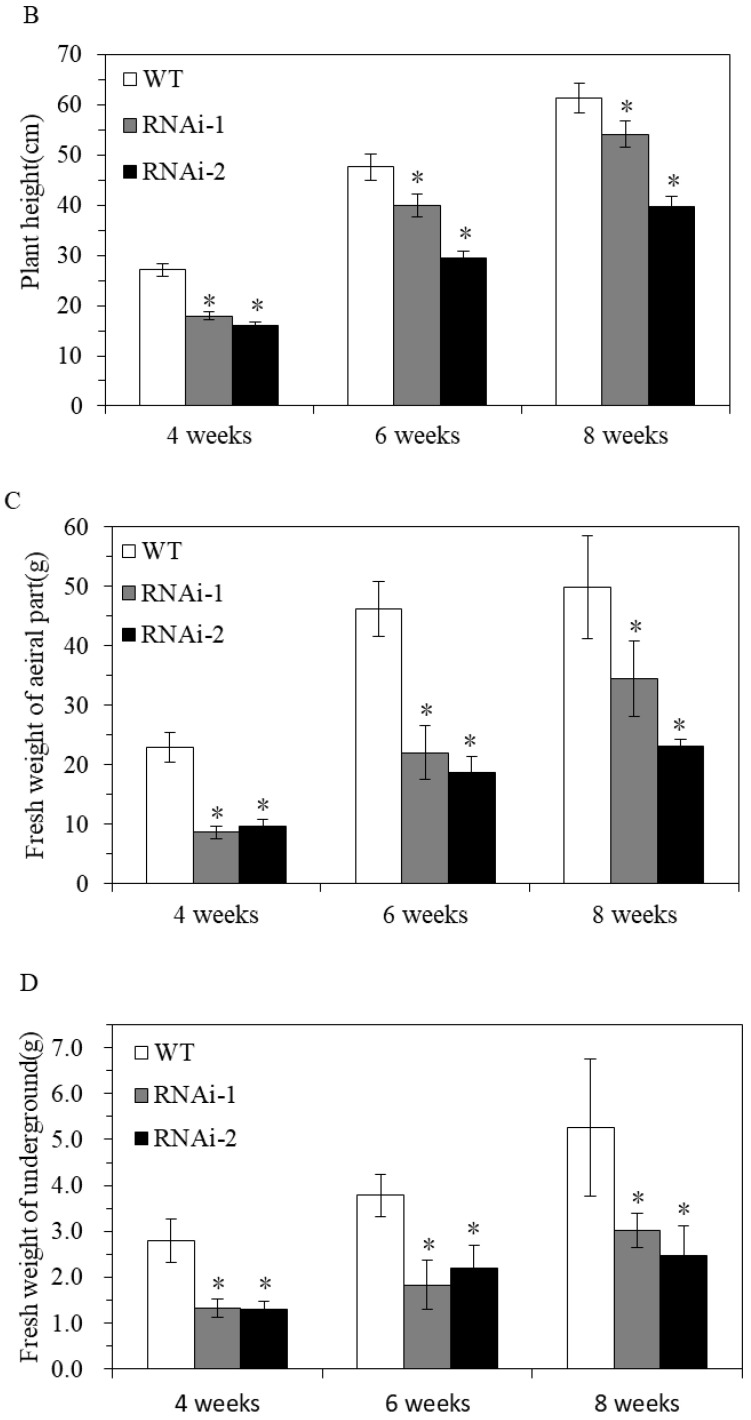
The phenotype of StSUT2-RNA interference plants (**A**) and its plants height (**B**) and fresh weight of the aerial (**C**) and underground parts (**D**). Plants were monitored within 4 weeks, 6 weeks, and 8 weeks of time duration. “*” means the difference is statistically significant between RNAi lines and WT.

**Figure 5:**
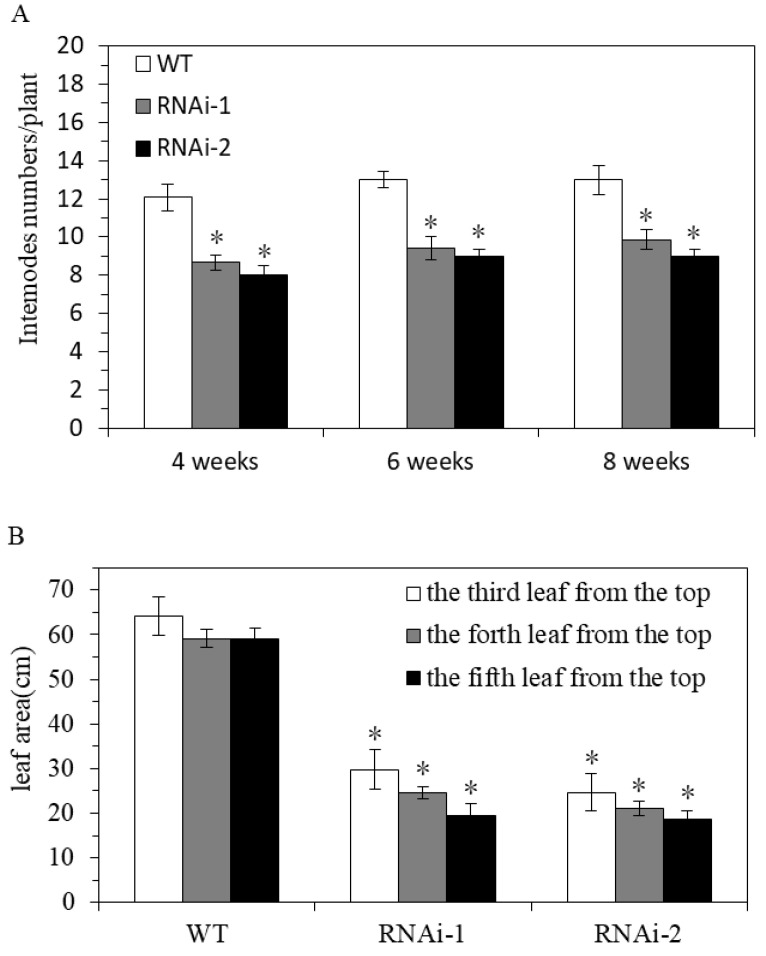
StSUT2-RNAi interference negatively affects the internode numbers (**A**) and leaf area (**B**). Leaf samples were taken at the third, fourth, and fifth leaves from the top of the plants. “*” means the difference is statistically significant between RNAi lines and WT.

To clarify the effect of StSUT2-RNA interference (StSUT2-RNAi) on potatoes, vegetative growth, plant height, fresh weight, internode numbers, and leaf area were monitored after 4 weeks, 6 weeks, and 8 weeks of the culture conditions. At the indicated times, the plant height, fresh weight, and tubers were measured, and the results indicated that the plant heights of all the samples consistently increased. However, the plant height of the wild-type line was 7.2 cm higher than RNAi-1 and 21.8 cm higher than RNAi-2 at the 8th week. The same difference was also noticed in the fresh weight of the aerial parts of the plants, where the wild-type aerial parts measured 15.2 g higher than RNAi-1 and 26.5 g more than RNAi-2 at the 8th week. A similar finding was noticed with the tuber weight of the wild-type measuring 2.2 g higher than the RNAi-1 line and 2.7 g noticeably greater than the RNAi-2 line. StSUT2-RNAi plants had substantially reduced plant heights throughout their development compared to the wild-type (WT) (Figure 4A,B). The fresh weight of aerial and underground part in StSUT2-RNAi plants also significantly decreased compared with the WT plants (Figure 4C,D). The StSUT2-RNAi plants showed few internode numbers than the WT in all growth culture periods (Figure 5A). A constant increase in the number of internodes was observed with each sample. However, the WT line had approximately four more internodes than the interference line each time of testing. A similar pattern was also observed with the leaf area measured in cm^2^. The WT line top leaf surface area was 34.2, 34.5, and 39.7 cm^2^ larger than the third, fourth, and fifth RNAi-1 top leaf surface areas, respectively, two times larger than the RNAi-1 and RNAi-2 leaves surface area. The observation was done at the third, fourth, and fifth leaves from the tops of the cultivated plants, which consistently displayed a StSUT2-RNAi leaf area smaller than that of the wild-type plants (Figure 5B).

#### 3.2.2. Effect of StSUT2-RNAi on Flowering and Tuber Yield

**Figure 6:**
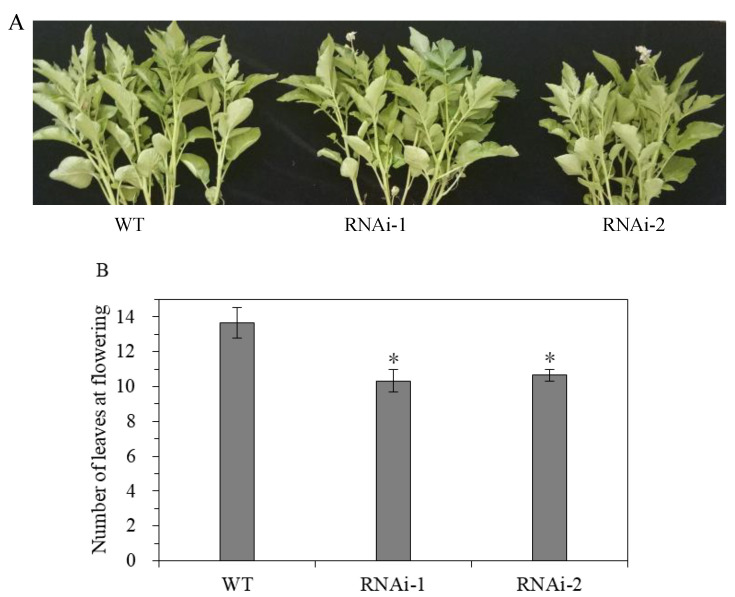
Flowering time (**A**) and the number of leaves at the flowering time (**B**) are significantly affected by StSUT2-RNAi. “*” means the difference is statistically significant between RNAi lines and WT.

**Figure 7:**
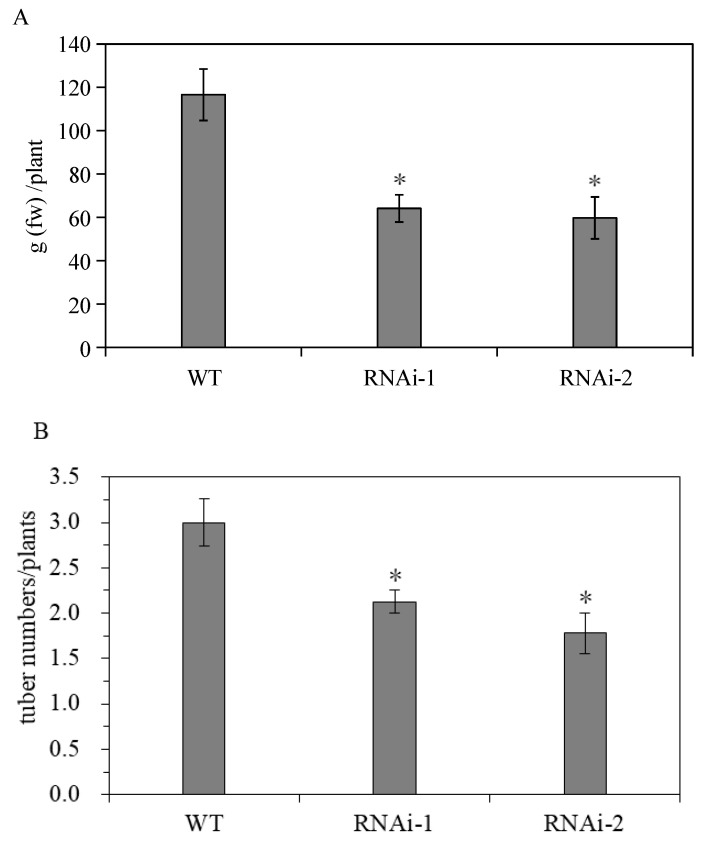

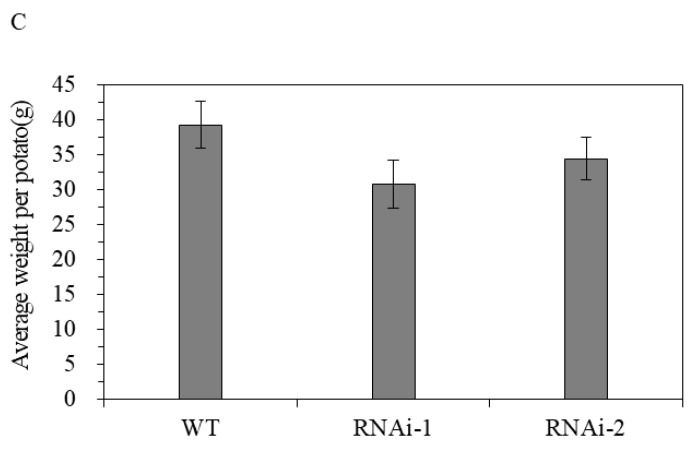
Effects of StSUT2-RNAi on potato tuber yield (**A**), number (**B**), and size (**C**). “*” means the difference is statistically significant between RNAi lines and WT.

The results show that StSUT2-RNAi affects the flowering time and tuber yield during potato ‘plant’ growth and development. StSUT2-RNAi plants flowered 4 to 6 days earlier than that of the WT (Figure 6A). In addition, the number of leaves at the flowering time of the StSUT2-RNAi plants was significantly less than that of the WT plants (Figure 6B). The analysis showed that there were 3 to 4 leaves more leaves by the WT plants in comparison with the StSUT2-RNAi. The average yield of the harvest tubers from the WT potato plants was 116.9 g, while the yields from the StSUT2-RNAi-1 and -2 potato plants were 64.1 g and 59.7 g, respectively, almost two times less in comparison to the WT potato yield. The StSUT2-RNAi plants displayed a strong reduction in tuber yield and numbers compared to the WT plants (Figure 7A,B). In contrast to the yield and tuber numbers, there was no significant difference in the average weight of a single tuber found between the WT and StSUT2-RNAi plants at maturity (Figure 7C). The average weight of a single potato tuber in the WT, RNAi-1, and RNAi-2 plants was 39.3 g, 30.7 g, and 34.4 g, respectively.

#### 3.2.3. Effect of StSTU2-RNAi on Carbohydrate Content

Sucrose transporters are essential for sugar accumulation in plants. To determine whether the levels of the starch content and other soluble sugars: glucose, sucrose, and fructose in leaves and tubers were modified by the interference of StSUT2, the above metabolites were analyzed. However, our data showed that the starch level and soluble sugar concentrations in the leaves and tubers were not significantly different between the StSUT2-RNAi and WT plants (Figures S1 and S2), which indicated that StSUT2 is not involved in carbohydrates accumulation in potato plants.

### 3.3. RNA-Seq Transcriptome Analysis between StSUT2-RNAi and WT Plants

**Figure 8:**
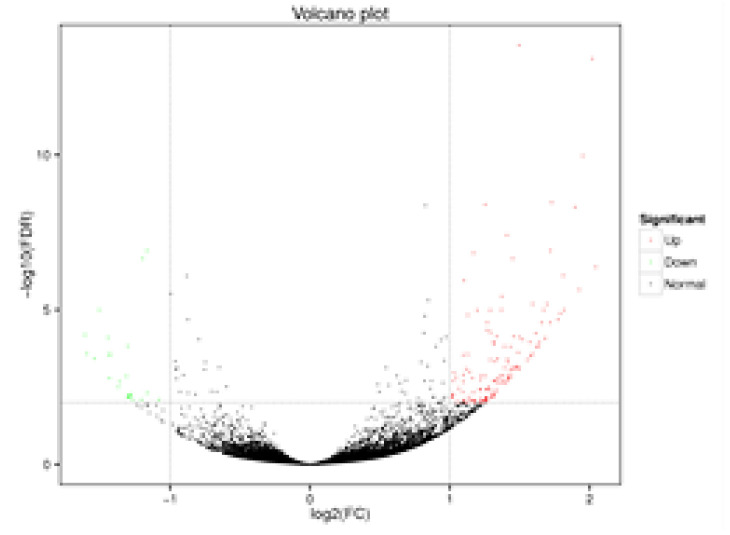
Volcano plot of differentially expressed gene distribution.

**Figure 9:**
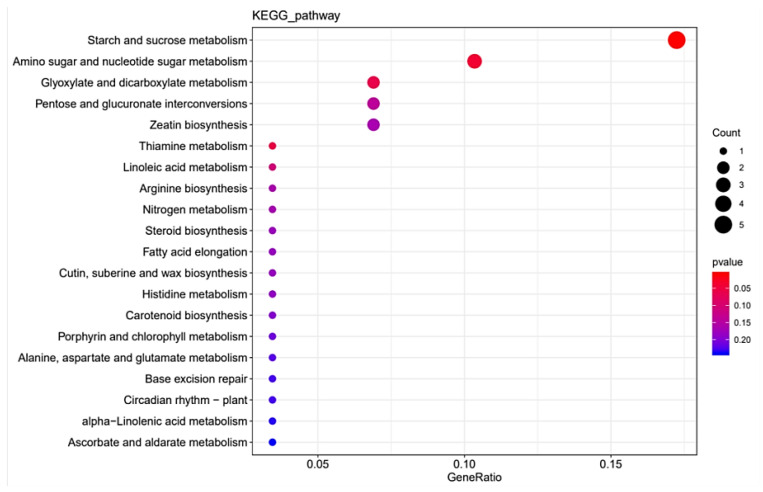
The enriched KEGG pathway of differentially expressed genes.

**Figure 10:**
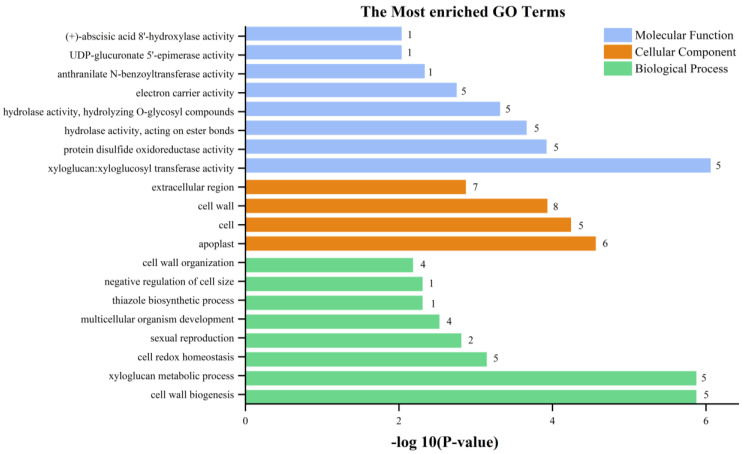
The most enriched GO terms of differentially expressed genes.

The RNA-seq data provides a foundation for elucidating the differentially expressed genes (DEGs) between StSUT2-RNAi-2 and WT plants. In total, 152 DEGs were detected, with 128 upregulated and 24 downregulated genes with a fold change of  ≥2 or  ≤− 2 relative to the WT (Figure 8 and Table S2). To explore the possible functions of the DEGs, the gene ontology (GO) and Kyoto Encyclopedia of Genes and Genomes (KEGG) databases were performed. The KEGG pathway class analysis of the DEGs revealed that the following two pathways were significantly enriched (*p* < 0.05): starch and sucrose metabolism and amino acid and nucleotide sugar metabolism (Figure 9, Table S3). The enriched KEGG pathway “starch and sucrose metabolism” has five genes: PGSC0003DMG400020589 (UDP-glucuronate 4-epimerase 6), PGSC0003DMG400011222 (UDP-glucuronate 4-epimerase 1), PGSC0003DMG400015933 (pectinesterase/pectinesterase inhibitor PPE8B-like), PGSC0003DMG400031816 (pectinesterase/pectinesterase inhibitor 41), and PGSC0003DMG400029738 (β-xylosidase/alpha-L-arabinofuranosidase 2), whereas the enriched KEGG pathway “amino acid and nucleotide sugar metabolism” has three genes: PGSC0003DMG400011222, PGSC0003DMG40002973, and PGSC0003DMG400020589, and they overlap with the three genes from the “starch and sucrose metabolism” pathway. Interestingly, these genes are totally involved in cell wall composition metabolism (Figure 9, Table 1). By the GO enrichment analysis, the 20 most significantly enriched terms (*p* < 0.01) are shown in Figure 10 (Table S4). After functional annotation, the DEGs in the nine terms are also involved in cell wall composition metabolism: “xyloglucan:xyloglucosyl transferase activity”, “hydrolase activity”, “hydrolyzing O-glycosyl compounds”, “UDP-glucuronate 5’-epimerase activity”, “apoplast”, “cell wall”, “extracellular region”, “cell wall organization”, “xyloglucan metabolic process”, and “cell wall biogenesis” (Figure 10). In total, 20 genes among 152 DEGs are relative to the cell wall composition metabolism; among them, 10 DEG expression levels were detected by qPCR in StSUT2-RNAi potato plants in order to test the accuracy of the RNA-seq data, which showed the express mode is similar between RNA-seq and qPCR, although the change fold is different (Table 1). The above results have indicated that the reduction of StSUT2 expression has caused changes in the gene expression levels of the cell wall composition metabolism.

## 4. Discussion

### 4.1. StSUT2 Is Involved in Different Physiological Characteristics of Potato Plants

It has been shown that StSUT2 mRNA and proteins are colocalized with StSUT1 and StSUT4 [6]. Our study confirmed that the expression levels of StSUT2, StSUT1, and StSUT4 are different in the stem, sink leaf, source leaf, roots, and tubers of potato plants (Figure 2). StSUT2 is highly expressed in sink leaf, similar to its ortholog in tomatoes [26], with a lower expression in other tissues. This study was designated to demonstrate the role of StSUT2 in potato plants’ vegetative growth, flowering, and tuber formation by analyzing the various physiological parameters due to StSUT2-RNA interference (Figure 4 and Figure 5). The interference of StSUT2 in potatoes significantly affects the plant height (Figure 4), similar to the SlSUT2 RNAi tomato plants with a dwarfed phenotype [27]. In addition, these results correlated with the previous findings in rice, which revealed that OsSUT2 is involved in Suc transport across the tonoplast from the vacuole lumen to the cytosol in rice, facilitating the transport of sugar from the source leaves to sink organs [28]. Moreover, another study confirmed that LeSUT2 is required for pollen tube development and germination, which impacts the tomato (*Lycopersicon esculentum*) plant fruit yield [15]. It was found that SlSUT2 directly interacts with the brassinosterioid (BR) signaling pathway components, namely the BR coreceptor BAK1 and the BR signaling inhibitor MSBP1 [29]. Therefore, a dwarfed growth behavior in SUT2 RNAi tomato and potato plants appears to be due to a defective BR signal pathway. StSUT2-RNAi potato plants show early flowering compared with the wild-type (Figure 6), similar to StSUT4-RNAi potato plants. The StSUT2-RNAi potato plants were also accompanied by a significant reduction in the tuber yield and tuber number per plant (Figure 7), where the number of fruits per plant was slightly increased. Still, the total fruit yield was slightly lower, indicating smaller fruits in SlSUT2-RNAi tomato plants. Potato tubers are produced through the vegetative growth of lateral underground buds that form at the base of the main stem, and they are usually accompanied by flowering, whereas tomato fruits are reproductive organs; therefore, the function of SUT2 in the organs of vegetation and reproduction might be different.

### 4.2. Functional Annotation of Differentially Expressed Genes (DEGs)

Previous studies have shown RNA-seq as a powerful and cost-efficient tool for mining gene resources and functions. RNA-Seq analysis revealed that 152 genes were significantly differential expressed, including 128 upregulated and 24 downregulated genes (Figure 8, Table S2). The GO and KEGG analyses revealed that most of the DEGs in RNAi plants were associated with the metabolism of starch, sucrose, and xyloglucan. Furthermore, functional annotation of the DEGs identified many candidate genes that are mainly related to synthetizing the cell wall composition, such as xyloglucan endotransglucosylase and pectin acetylesterase and UDP-glucuronate 4-epimeraseand, and cell wall components such as arabinogalactan protein and expansin-like protein (Table 1). It gave a theoretical foundation for an improved understanding of the molecular mechanisms of StSUT2 involved in cell wall composition metabolism.

## 5. Conclusions

In conclusion, a comparative analysis of StSUT1, StSUT2, and StSUT4 revealed a relative difference in the expression in the stem, sink leaf, source leaf, root, and tubers of potato plants. By analysis of the phenotype characterizations of StSUT2-RNAi compared to WT potato plants, it revealed that StSUT2 interference significantly affects plant height, fresh weight, internodes number and leaf area, flowering time, and tuber yield. The functional annotation results of differentially expressed genes (DEGs) between StSUT2-RNAi transgenic and wild-type plants showed that DEGs were mainly related to the cell wall composition metabolism.

## Figures and Tables

**Table 1:** Differentially expressed genes on cell wall composition metabolism.

Gene ID	Annotation	RNA-Seq Fold Change	Regulation	RT-PCR Fold Change
PGSC0003DMG400021877	Xyloglucan endotransglucosylase/hydrolase 2	3.11	up	1.98
PGSC0003DMG400026189	Xyloglucan endotransglucosylase/hydrolase protein 9	3.01	up	2.09
PGSC0003DMG400004670	Xyloglucan endotransglucosylase/hydrolase 2	2.72	down	
PGSC0003DMG400000408	Xyloglucan endotransglucosylase/hydrolase protein 31	2.66	up	
PGSC0003DMG400004109	Xyloglucan endotransglucosylase/hydrolase protein 33	2.99	up	1.47
PGSC0003DMG400031731	ω-hydroxypalmitate O-feruloyl transferase	2.47	down	
PGSC0003DMG400016249	ω-hydroxypalmitate O-feruloyl transferase	3.12	up	
PGSC0003DMG400020589	UDP-glucuronate 4-epimerase 6	2.35	up	
PGSC0003DMG400011222	UDP-glucuronate 4-epimerase 1	2.54	up	1.49
PGSC0003DMG400023732	UDP-glucoronosyl and UDP-glucosyl transferase	2.29	down	1.33
PGSC0003DMG400029738	β-xylosidase/alpha-L-arabinofuranosidase 2	2.17	up	
PGSC0003DMG400000827	Galacturonosyltransferase-like 3	2.21	up	
PGSC0003DMG401024140	Pectin acetylesterase 8-like	2.33	up	1.23
PGSC0003DMG400015933	pectinesterase/pectinesterase inhibitor PPE8B-like	2.33	up	1.74
PGSC0003DMG400031816	Pectinesterase/pectinesterase inhibitor 41	2.87	up	2.53
PGSC0003DMG400026220	Expansin-like B1	2.91	down	
PGSC0003DMG400018635	Expansin-like A1	2.68	up	1.15
PGSC0003DMG400014408	Fasciclin-like arabinogalactan protein 1	2.25	up	
PGSC0003DMG402013388	Classical arabinogalactan protein 4-like	2.51	down	
PGSC0003DMG401027116	Laccase-12	3.05	down	2.11

## Data Availability

The data presented in this study are available on request from the corresponding author.

## Supplementary Materials

The following supporting information can be downloaded at: https://www.mdpi.com/article/10.3390/cimb45030172/s1. Figure S1: Carbohydrate content in leaves, Figure S2: Carbohydrate content in tubers, Table S1: Primers sequence, Table S2: 152 differentially expressed genes (DEGs) between StSUT2 RNAi line2 and WT, Table S3: The enriched KEGG pathway of differentially expressed genes between StSUT2 RNAi line2 and WT. Table S4: The most enriched GO terms of differentially expressed genes between StSUT2 RNAi line2 and WT.
