# Supplementary material for: Sucrose Transporter StSUT2 Affects Potato Plants Growth, Flowering Time, and Tuber Yield
Source: Curr Issues Mol Biol. 2023 Mar 22;45(3):2629–43. doi: 10.3390/cimb45030172 (PMC10047837; doi:10.3390/cimb45030172)
Supplement: Supplementary file 1 [file cimb-45-00172-s001.zip › cimb-2186099-supplementary.pdf]

Table S1. Primers sequence.

Genes ID/name	Gene description	Forward primer	Reverse primer
StSUT1	Sucrose transporter 1 in *Solanum tuberosum*	TTCCATAGCTGCTGGTG TTC	TACCAGAAATGGGTCC ACAA
StSUT2	Sucrose transporter 2 in *Solanum tuberosum*	GGCATTCCTCTTGCTGTA ACC	GCGATACAACCATCTG AGGGTAC
StSUT4	Sucrose transporter 4 in *Solanum tuberosum*	GCTCTTGGGCTTGGACA AGGC	GGCTGGTGAATTGCCT CCACC
ef1α	*ef1α in Solanum tuberosum*	CAAGGATGACCCAGCC AAG	TTCCTTACCTGAACGC CTGT
PGSC0003DMG400021877/StXT H2	Xyloglucan endotransglucosylase/hydrolase 2	AATGCTGATGATTGGGC CACA	AGCTTTGGGCCATGTA GTTTC
PGSC0003DMG400026189/StXT H9	Xyloglucan endotransglucosylase/hydrolase protein 9	AGATGAGACCCCTGTTC GTGT	CGCCTTGTGTAGCCCA ATCAT
PGSC0003DMG400004109/StXT H33	Probable xyloglucan endotransglucosylase/hydrolase protein 33	CAGGATTGGATTGGGCT AGGA	CACGGTATGCTTTGTCC ACTA
PGSC0003DMG400011222/StG ALE	UDP-glucuronate 4-epimerase 1	TGTTTCCGTCTACACCTG GGA	TGCCCATAGAAACATC GTGCT
PGSC0003DMG400023732/StU GTs)	UDP-glucoronosyl and UDP-glucosyl transferase	TTGGGTAATACGTCTCC GGTG	CCAACATCGCAACCAT CATCG
PGSC0003DMG401024140/StPA E	Pectin acetylesterase 8-like	TATTTTCAGGGAGGAGG TTGG	CTGAGAATCCCTGAGA AAGCG
PGSC0003DMG400015933/StPP E8	Pectinesterase/pectinesterase inhibitor PPE8B-like	TCGATGGCACAAACGG ACTAG	GGGGCGGACCATAAG AAGAAT
PGSC0003DMG400031816/StPE I	Probable pectinesterase/pectinesterase inhibitor 41	GTAGGCCATGGAAAGA GTACT	AATCTCCAGACCAATC GTGCC
PGSC0003DMG400018635/StEX P	Expansin-like A1	CAGTGTTGATGTTGCTC AGGT	AGTTCCAGTTCGACGA TCCAA
PGSC0003DMG401027116/StL AC12	Laccase-12-like	CAAGCACATCACCAGG GAATA	AAAGAGGTTGCCAGA GTGTCC

Table S2. 152 differentially expressed genes (DEGs) between StSUT2 RNAi line2 and WT. Genes with log2 fold change ≥ 1 with P value < 0.01 were regarded as DEGs. Three biological replicates were averaged.

Gene ID	Functional annotation	Log_2 fold change	Fold change	FDR p-value
PGSC0003DMG400000386	Leucine-rich repeat extensin-like protein 3	1.312830164	2.484284093	0.002008144
PGSC0003DMG400000408	Xyloglucan endotransglucosylase/hydrolase protein 31	1.411889279	2.660853865	0.000403251
PGSC0003DMG400000818	Lysine histidine transporter-like 6	1.31550654	2.488897022	0.006029805
PGSC0003DMG400000827	Galacturonosyltransferase-like 3	1.496999317	2.822550351	0.000780393
PGSC0003DMG400000922	Myb family transcription factor	-1.295792877	-2.455118869	0.006209645
PGSC0003DMG400001417	Homeobox-leucine zipper protein HAT7	1.269952647	2.411536501	0.007428824
PGSC0003DMG400001462	Protein LONGIFOLIA 1	1.26361126	2.400959819	0.006668159
PGSC0003DMG400001598	Snakin-2	1.554435931	2.937188652	0.000418436
PGSC0003DMG400001634	Protein FAF-like	1.128717441	2.186642608	0.000274496
PGSC0003DMG400001830	Receptor-like protein 9DC3	1.499805063	2.828044973	0.000773618
PGSC0003DMG400001990	Putative late blight resistance protein homolog	1.951139293	3.866797712	1.06E-10
PGSC0003DMG400002420	Elongation of fatty acids protein 3-like	1.925881132	3.799688451	2.26E-06
PGSC0003DMG400002519	GDSL esterase/lipase APG	1.413820921	2.6644189	0.000495133
PGSC0003DMG400002883	Axial regulator YABBY 5	1.671125157	3.184628657	1.00E-05
PGSC0003DMG400002999	Protein FAF-like	1.323746401	2.503152871	0.000468424
PGSC0003DMG400003188	Serine carboxypeptidase	1.023386864	2.032685285	0.006372868
PGSC0003DMG400003522	Galactinol synthase 2	1.37511104	2.593878745	0.001361314
PGSC0003DMG400004109	Xyloglucan endotransglucosylase/hydrolase protein 33	1.577948947	2.985451118	0.000318645

PGSC0003DMG400004252	Protein STAY-GREEN	-1.362133991	-2.570651414	0.002119065
PGSC0003DMG400004670	Xyloglucan endotransglucosylase/hydrolase 2	-1.444254069	-2.721220889	7.90E-05
PGSC0003DMG400004957	Unknown Function	1.480198893	2.789871924	0.000780393
PGSC0003DMG400005130	Unknown Function	1.217829074	2.325964494	0.007424572
PGSC0003DMG400005156	Putative F-box protein	-1.302334791	-2.466276907	0.000150016
PGSC0003DMG400005688	Regulator of Vps4 activity in the MVB pathway	1.369010644	2.582933757	1.00E-05
PGSC0003DMG400006890	Unknown Function	1.271171441	2.413574637	0.003717846
PGSC0003DMG400007239	BTB/POZ domain-containing protein DOT	1.443473934	2.719749791	0.000780393
PGSC0003DMG400007244	GDSL esterase/lipase	1.682522116	3.209886123	8.39E-05
PGSC0003DMG400007385	Disease resistance protein	1.303851989	2.468871911	0.006607122
PGSC0003DMG400007972	Abscisic acid 8- hydroxylase	1.267981232	2.408243433	4.54E-05
PGSC0003DMG400008021	Protein phosphatase 2C	1.408661114	2.654906612	0.002028627
PGSC0003DMG400008110	Protein indeterminate-domain 9	1.254011688	2.385037067	0.008622626
PGSC0003DMG400008224	Glutaredoxin-C6	1.013193684	2.018374219	0.006613075
PGSC0003DMG400008381	Unknown Function	1.818287137	3.526622459	1.00E-05
PGSC0003DMG400009888	Mechanosensitive ion channel protein 8	1.123023019	2.178028782	0.009859787
PGSC0003DMG400009985	Serine/threonine-protein kinase PIX13	1.196659987	2.292084112	1.02E-05
PGSC0003DMG400010158	Kinase-interacting family protein	1.789971585	3.458080815	1.32E-05
PGSC0003DMG400010406	Unknown Function	1.441807531	2.716610124	0.001414627
PGSC0003DMG400010762	Monothiol glutaredoxin-S1	1.657326663	3.154314833	2.63E-05
PGSC0003DMG400010764	Monothiol glutaredoxin-S1	1.695575104	3.239059801	6.54E-06
PGSC0003DMG400010798	Staygreen protein	-1.269914382	-2.41147254	0.0081065
PGSC0003DMG400010819	3-ketoacyl-CoA synthase 1	1.475279859	2.780375734	0.000673583
PGSC0003DMG400010870	NAC domain-containing protein 22	1.290497351	2.44612368	0.000418436
PGSC0003DMG400011133	Transcription factor bHLH66	1.044018565	2.061963171	0.00151353
PGSC0003DMG400011222	UDP-glucuronate 4-epimerase 1	1.346089027	2.542220251	0.002366968
PGSC0003DMG400011230	Unknown Function	1.252716548	2.382896926	4.05E-09
PGSC0003DMG400011287	Methyladenine glycosylase	1.459711014	2.750532622	0.000780393
PGSC0003DMG400012195	Unknown Function	1.290676511	2.446427469	5.42E-05
PGSC0003DMG400012316	Respiratory burst oxidase homolog protein A	2.01922578	4.053661944	7.72E-14
PGSC0003DMG400012479	Protein NRT1/ PTR FAMILY 6.3	1.489561035	2.808035228	0.000240715
PGSC0003DMG400012547	ABC transporter G family member 11	1.065533507	2.092943705	0.00350311
PGSC0003DMG400012641	BAG family molecular chaperone regulator 3	1.315485429	2.488860602	0.006029805
PGSC0003DMG400013549	Auxin-responsive protein SAUR72	1.315912137	2.489596845	0.0001282
PGSC0003DMG400014408	Fasciclin-like arabinogalactan protein 1	1.16895055	2.248480777	0.004772605
PGSC0003DMG400014621	Transcription factor bHLH153	1.257227363	2.390359091	0.003877606
PGSC0003DMG400014850	14 kDa proline-rich protein DC2.15	1.718174711	3.290198695	2.63E-05
PGSC0003DMG400014869	Formin-like protein 6	1.170736987	2.251266714	0.009290829
PGSC0003DMG400015194	Malate synthase, glyoxysomal	1.142115272	2.207043817	0.001012258
PGSC0003DMG400015523	Chitinase 2	1.096226384	2.137947437	0.000289299
PGSC0003DMG400015933	Pectinesterase/pectinesterase inhibitor PPE8B	1.220586446	2.330414278	0.003868046
PGSC0003DMG400016032	Monothiol glutaredoxin-S2	1.33065011	2.51515988	0.003989929
PGSC0003DMG400016033	Monothiol glutaredoxin-S	1.497551983	2.823631817	7.79E-05
PGSC0003DMG400016249	ω-hydroxypalmitate O-feruloyl transferase	1.640399314	3.117521077	0.000112599
PGSC0003DMG400016608	Transcription factor bHLH62	1.810738632	3.508218566	7.89E-07
PGSC0003DMG400016828	Protein EXORDIUM-like 5	1.77408541	3.420211188	2.07E-05
PGSC0003DMG400016915	Non-symbiotic hemoglobin 2	1.029906881	2.041892453	0.001087283
PGSC0003DMG400017291	LRR receptor-like serine/threonine-protein kinase	1.614662825	3.062400191	5.74E-05
PGSC0003DMG400017373	Unknown Function	1.338378416	2.528669375	0.001649108
PGSC0003DMG400017763	Serine/threonine-protein kinase SAPK2	1.421509297	2.67865596	0.001697384
PGSC0003DMG400018128	Unknown Function	1.179836434	2.265510903	0.009159219
PGSC0003DMG400018220	PCTP-like protein	1.25272903	2.382917543	0.007307961
PGSC0003DMG400018635	Expansin-like A1	1.423433528	2.682231069	0.001418976
PGSC0003DMG400018641	24-methylenesterol C-methyltransferase 2	1.223211048	2.334657709	0.001372518
PGSC0003DMG400018884	Calcium-binding protein KIC	1.16953749	2.249395726	1.49E-07
PGSC0003DMG400019212	DELLA protein GAI	1.391725104	2.62392249	0.000761076
PGSC0003DMG400019257	Thiamine thiazole synthase 1	1.313246581	2.485001256	0.000108727
PGSC0003DMG400019432	Unknown Function	1.100019172	2.143575411	0.0081065
PGSC0003DMG400019861	Transcription factor bHLH137	1.728469864	3.313761712	3.42E-09
PGSC0003DMG400020589	UDP-glucuronate 4-epimerase 6	1.232965193	2.350495947	0.009290829
PGSC0003DMG400021512	Acyl-CoA-binding domain-containing protein 3	-1.164027989	-2.240821901	0.004908583

PGSC0003DMG400021798	Unknown Function	1.895265418	3.719904072	4.78E-09
PGSC0003DMG400021877	Xyloglucan endotransglucosylase/hydrolase 2	1.636585755	3.109291236	0.000150016
PGSC0003DMG400021886	Unknown Function	1.219473172	2.328616678	0.001812988
PGSC0003DMG400022294	Glycerophosphodiester phosphodiesterase GDPDL4	1.183189118	2.270781854	0.009066877
PGSC0003DMG400022312	GDSL esterase/lipase	1.022504795	2.031442874	0.005277012
PGSC0003DMG400022371	Keratin	1.374607748	2.592974014	0.002281401
PGSC0003DMG400022555	Ubiquitin-conjugating enzyme	1.252071933	2.381832454	0.001405589
PGSC0003DMG400022677	CASP-like protein 1E2	1.389862627	2.620537269	0.000154138
PGSC0003DMG400022990	Receptor-like protein EIX2	1.328383363	2.51121119	0.002005927
PGSC0003DMG400023144	Aspartyl protease family protein	1.096091591	2.137747695	0.002160494
PGSC0003DMG400023458	Phenylalanine ammonia-lyase	-1.206178049	-2.307255941	0.0081065
PGSC0003DMG400023508	Receptor-like protein kinase HERK 1	1.335106804	2.522941586	7.33E-05
PGSC0003DMG400023620	Glutamine synthetase cytosolic isozym	1.408192296	2.654044014	3.90E-08
PGSC0003DMG400023732	UDP-glucoronosyl and UDP-glucosyl transferase	-1.196907505	-2.29247739	2.23E-07
PGSC0003DMG400024447	MDIS1-interacting receptor like kinase 2	1.097833509	2.140330383	0.001283576
PGSC0003DMG400024575	TORTIFOLIA1-like protein 3	1.298314484	2.459413788	0.00641985
PGSC0003DMG400024875	Unknown Function	1.457586939	2.746486002	7.20E-05
PGSC0003DMG400025030	Receptor-like protein kinase THESEUS 1	1.620442104	3.074692437	0.000157582
PGSC0003DMG400025102	Proline-rich receptor-like protein kinase PERK10	1.091171138	2.130469116	0.004675913
PGSC0003DMG400025336	Protein NRT1/ PTR FAMILY 2.11	1.163251626	2.239616363	0.001641716
PGSC0003DMG400025587	ATP-citrate synthase alpha chain protein 2	1.264428381	2.402320073	0.009006742
PGSC0003DMG400025843	Uncharacterized acetyltransferase	-1.280427073	-2.429108738	0.006372868
PGSC0003DMG400025867	LRR receptor-like serine/threonine-protein kinase	1.052530608	2.074164918	0.009290829
PGSC0003DMG400026189	Xyloglucan endotransglucosylase/hydrolase protein 9	1.590714553	3.011984933	0.000268587
PGSC0003DMG400026220	Expansin-like B1	-1.541201501	-2.91036783	0.000380367
PGSC0003DMG400026224	Non-symbiotic hemoglobin 2	1.591645902	3.013929983	6.54E-06
PGSC0003DMG400026289	Protein ENHANCED DISEASE RESISTANCE 2	1.432511642	2.699162138	0.000157582
PGSC0003DMG400026300	Histidine decarboxylase	-1.434340724	-2.702586369	0.001614153
PGSC0003DMG400026750	Serine/threonine-protein kinase BRI1-lik	1.340836212	2.532980924	0.000383216
PGSC0003DMG400027340	P450 77A1 precursor	1.495455478	2.819531533	2.80E-14
PGSC0003DMG400027839	Unknown Function	-1.4314296	-2.697138487	0.000268587
PGSC0003DMG400027916	Inositol-3-phosphate synthase	1.371675712	2.58770958	3.76E-06
PGSC0003DMG400028026	Protein NRT1/ PTR FAMILY 6.1	1.57291234	2.975046752	0.000112599
PGSC0003DMG400029738	β-xylosidase/alpha-L-arabinofuranosidase 2	1.11641485	2.168075279	0.000833426
PGSC0003DMG400030640	Unknown Function	1.23749704	2.357891021	0.008677362
PGSC0003DMG400030808	Protein EXORDIUM-like	1.371868015	2.58805453	0.002005927
PGSC0003DMG400030826	Protein EXORDIUM-like	1.399549739	2.638192321	0.002280477
PGSC0003DMG400031420	Cytokinin dehydrogenase 7	1.268113729	2.408464616	2.63E-05
PGSC0003DMG400031731	ω-hydroxypalmitate O-feruloyl transferase	-1.305117427	-2.471038394	0.006613075
PGSC0003DMG400031816	Pectinesterase/pectinesterase inhibitor 41	1.521324552	2.870544761	9.06E-05
PGSC0003DMG400032155	Linoleate 13S-lipoxygenase 2-1	1.106646259	2.153444675	0.009838253
PGSC0003DMG400032510	L-ascorbate oxidase	1.27938079	2.427347719	0.003235019
PGSC0003DMG400032534	Unknown Function	1.408890164	2.655328152	0.001446829
PGSC0003DMG400032817	Squamosa promoter-binding protein 1	2.044051903	4.12402164	4.21E-07
PGSC0003DMG400033590	Protein PELPK1	-1.294042118	-2.452141307	0.00640728
PGSC0003DMG400033591	Proteoglycan 4-like isoform X4	-1.285476887	-2.437626159	0.005288759
PGSC0003DMG400034309	Non-specific lipid transfer protein GPI-anchored 1	1.091160742	2.130453764	0.009808963
PGSC0003DMG400039158	Unknown Function	1.292447785	2.449432924	0.00426552
PGSC0003DMG401000895	Chlorophyllide a oxygenase	-1.300894884	-2.463816625	0.006372868
PGSC0003DMG401008665	Carboxylesterase 15	1.101858232	2.146309653	1.15E-06
PGSC0003DMG401009879	GDSL esterase/lipase	1.718898252	3.291849211	1.23E-07
PGSC0003DMG401014997	GDSL esterase/lipase	1.213185893	2.318490625	0.003802601
PGSC0003DMG401020115	Fimbrin-2-like	1.308250153	2.476409936	0.005650192
PGSC0003DMG401024140	Pectin acetylesterase 8-like	1.219755277	2.329072061	0.009634419
PGSC0003DMG401027116	Laccase-12	-1.609895509	-3.052297339	6.52E-05
PGSC0003DMG402008903	Cyclin-dependent protein kinase inhibitor SMR4	1.325355616	2.505946502	0.004731237
PGSC0003DMG402012350	Protein LURP-one-related 11	1.449064585	2.730309661	2.24E-07
PGSC0003DMG402012500	Homeobox-leucine zipper protein HAT4	1.36269052	2.571643251	0.001446829
PGSC0003DMG402013304	WEB family protein	-1.507094466	-2.842370195	1.01E-05
PGSC0003DMG402013388	Classical arabinogalactan protein 4-like	-1.370152147	-2.584978259	0.003165788
PGSC0003DMG402020132	Non-specific lipid-transfer protein	-1.596057608	-3.023160572	0.000263835

PGSC0003DMG402021252	Pentatricopeptide repeat-containing protein	1.682891219	3.210707454	8.39E-05
PGSC0003DMG402023438	Epoxide hydrolase	1.128498787	2.186311227	1.48E-05
Solanum_tuberosum_newGene_1801	3-hydroxybenzoate 6-hydroxylase 1-like	1.14280741	2.208102908	8.62E-03
Solanum_tuberosum_newGene_2357	Protein GIGANTEA-like isoform X1	1.289058725	2.443685672	0.0000102
Solanum_tuberosum_newGene_4227	Bidirectional sugar transporter SWEET12-like	1.133909553	2.194526289	6.67E-03
Solanum_tuberosum_newGene_4341	Unknown Function	1.164067387	2.240883096	0.008125597
Solanum_tuberosum_newGene_4360	Metallocarboxypeptidase inhibitor-like	-1.31242534	2.483587094	0.001414627
Solanum_tuberosum_newGene_4390	Inactive purple acid phosphatase 27	-1.081007719	2.115513246	8.11E-03
Solanum_tuberosum_newGene_4977	Protein SPA1-RELATED 4-like]	-1.432362059	2.698882296	2.92E-04
Solanum_tuberosum_newGene_6254	Inactive poly [ADP-ribose] polymerase RCD1	-1.165977224	2.243851537	0.000000123
Solanum_tuberosum_newGene_7994	Unknown Function	1.431227599	2.69676087	9.56E-04
Solanum_tuberosum_newGene_8481	Unknown Function	1.026453589	2.037010743	0.002008144

Table S3. The enriched KEGG pathway of differentially expressed genes between StSUT2 RNAi line2 and WT.

#Kegg_pathway	ko_id	KEGG_N	P-value	Gene_id
**Starch and sucrose metabolism**	ko00500	5	0.008738399	PGSC0003DMG400011222;PGSC0003DMG400015933; PGSC0003DMG400020589;PGSC0003DMG400029738; PGSC0003DMG400031816
Amino sugar and nucleotide sugar metabolism	ko00520	3	0.038830822	PGSC0003DMG400011222;PGSC0003DMG400020589; PGSC0003DMG400029738
Glyoxylate and dicarboxylate metabolism	ko00630	2	0.061510158	PGSC0003DMG400015194;PGSC0003DMG400023620
Pentose and glucuronate interconversions	ko00040	2	0.141156746	PGSC0003DMG400015933;PGSC0003DMG400031816
Zeatin biosynthesis	ko00908	2	0.163163085	PGSC0003DMG400023732;PGSC0003DMG400031420
Thiamine metabolism	ko00730	1	0.057062138	PGSC0003DMG400019257
Linoleic acid metabolism	ko00591	1	0.106935112	PGSC0003DMG400032155
Arginine biosynthesis	ko00220	1	0.161904319	PGSC0003DMG400023620
Nitrogen metabolism	ko00910	1	0.165703127	PGSC0003DMG400023620
Steroid biosynthesis	ko00100	1	0.177000067	PGSC0003DMG400018641
Cutin, suberine and wax biosynthesis	ko00073	1	0.180732785	PGSC0003DMG400031731
Fatty acid elongation	ko00062	1	0.180732785	PGSC0003DMG400010819
Histidine metabolism	ko00340	1	0.184449154	PGSC0003DMG400026300
Carotenoid biosynthesis	ko00906	1	0.19550086	PGSC0003DMG400007972
Porphyrin and chlorophyll metabolism	ko00860	1	0.210011746	PGSC0003DMG401000895
Alanine, aspartate and glutamate metabolism	ko00250	1	0.220728733	PGSC0003DMG400023620
Base excision repair	ko03410	1	0.227795259	PGSC0003DMG400011287
Circadian rhythm - plant	ko04712	1	0.227795259	Solanum_tuberosum_newGene_2357
alpha-Linolenic acid metabolism	ko00592	1	0.234799895	PGSC0003DMG400032155
Ascorbate and aldarate metabolism	ko00053	1	0.238279168	PGSC0003DMG400032510

Table 4. The most enriched GO terms of differentially expressed genes between StSUT2 RNAi line2 and WT.

GO_ID	GO_term	ontology	Items	p-value	Gene_ID

GO:0016762	xyloglucan:xyloglucosyl transferase activity	Molecular Function	5	8.77E-07	PGSC0003DMG400026189;PGSC0003DMG400021877;PGSC0003DMG400000408;PGSC0003DMG400004109;PGSC0003DMG400004670
GO:0015035	protein disulfide oxidoreductase activity	Molecular Function	5	0.000120647	PGSC0003DMG400010762;PGSC0003DMG400010764;PGSC0003DMG400016033;PGSC0003DMG400008224;PGSC0003DMG400016032
GO:0016788	hydrolase activity, acting on ester bonds	Molecular Function	5	0.000219419	PGSC0003DMG400007244;PGSC0003DMG401014997;PGSC0003DMG400022312;PGSC0003DMG400002519;PGSC0003DMG401009879
GO:0004553	hydrolase activity, hydrolyzing O-glycosyl compounds	Molecular Function	5	0.000486003	PGSC0003DMG400004109;PGSC0003DMG400021877;PGSC0003DMG400000408;PGSC0003DMG400004670;PGSC0003DMG400026189
GO:0009055	electron carrier activity	Molecular Function	5	0.001790711	PGSC0003DMG400008224;PGSC0003DMG400016032;PGSC0003DMG400016033;PGSC0003DMG400010764;PGSC0003DMG400010762
GO:0047672	anthranilate N-benzoyltransferase activity	Molecular Function	1	0.004670586	PGSC0003DMG400016249
GO:0050379	UDP-glucuronate 5'-epimerase activity	Molecular Function	1	0.009319614	PGSC0003DMG400011222
GO:0010295	(+)-abscisic acid 8'-hydroxylase activity	Molecular Function	1	0.009319614	PGSC0003DMG400007972
GO:0048046	apoplast	Cellular Component	6	2.75E-05	PGSC0003DMG400004670;PGSC0003DMG400021877;PGSC0003DMG400000408;PGSC0003DMG400004109;PGSC0003DMG401027116;PGSC0003DMG400026189
GO:0005623	cell	Cellular Component	5	5.79E-05	PGSC0003DMG400008224;PGSC0003DMG400016032;PGSC0003DMG400010762;PGSC0003DMG400016033;PGSC0003DMG400010764
GO:0005618	cell wall	Cellular Component	8	0.000117471	PGSC0003DMG400000408;PGSC0003DMG400015933;PGSC0003DMG400004109;PGSC0003DMG400001598;PGSC0003DMG400031816;PGSC0003DMG400026189;PGSC0003DMG400021877;PGSC0003DMG400004670
GO:0005576	extracellular region	Cellular Component	7	0.001356057	PGSC0003DMG400032510;PGSC0003DMG400001598;PGSC0003DMG400026220;PGSC0003DMG400021512;PGSC0003DMG400018635;PGSC0003DMG400015933;PGSC0003DMG400015523
GO:0042546	cell wall biogenesis	Biological Process	5	1.35E-06	PGSC0003DMG400004670;PGSC0003DMG400004109;PGSC0003DMG400021877;PGSC0003DMG400026189;PGSC0003DMG400000408
GO:0010411	xyloglucan metabolic process	Biological Process	5	1.35E-06	PGSC0003DMG400000408;PGSC0003DMG400026189;PGSC0003DMG400021877;PGSC0003DMG400004109;PGSC0003DMG400004670
GO:0045454	cell redox homeostasis	Biological Process	5	0.000726648	PGSC0003DMG400010762;PGSC0003DMG400010764;PGSC0003DMG400016033;PGSC0003DMG400016032;PGSC0003DMG400008224
GO:0019953	sexual reproduction	Biological Process	2	0.00155711	PGSC0003DMG400018635;PGSC0003DMG400026220
GO:0007275	multicellular organism development	Biological Process	4	0.003001516	PGSC0003DMG400033590;PGSC0003DMG400002883;PGSC0003DMG400007972;PGSC0003DMG400033591
GO:0052837	thiazole biosynthetic process	Biological Process	1	0.004966194	PGSC0003DMG400019257
GO:0045792	negative regulation of cell size	Biological Process	1	0.004966194	PGSC0003DMG400010870
GO:0071555	cell wall organization	Biological Process	4	0.006619091	PGSC0003DMG400021877;PGSC0003DMG400000408;PGSC0003DMG400004670;PGSC0003DMG400026189

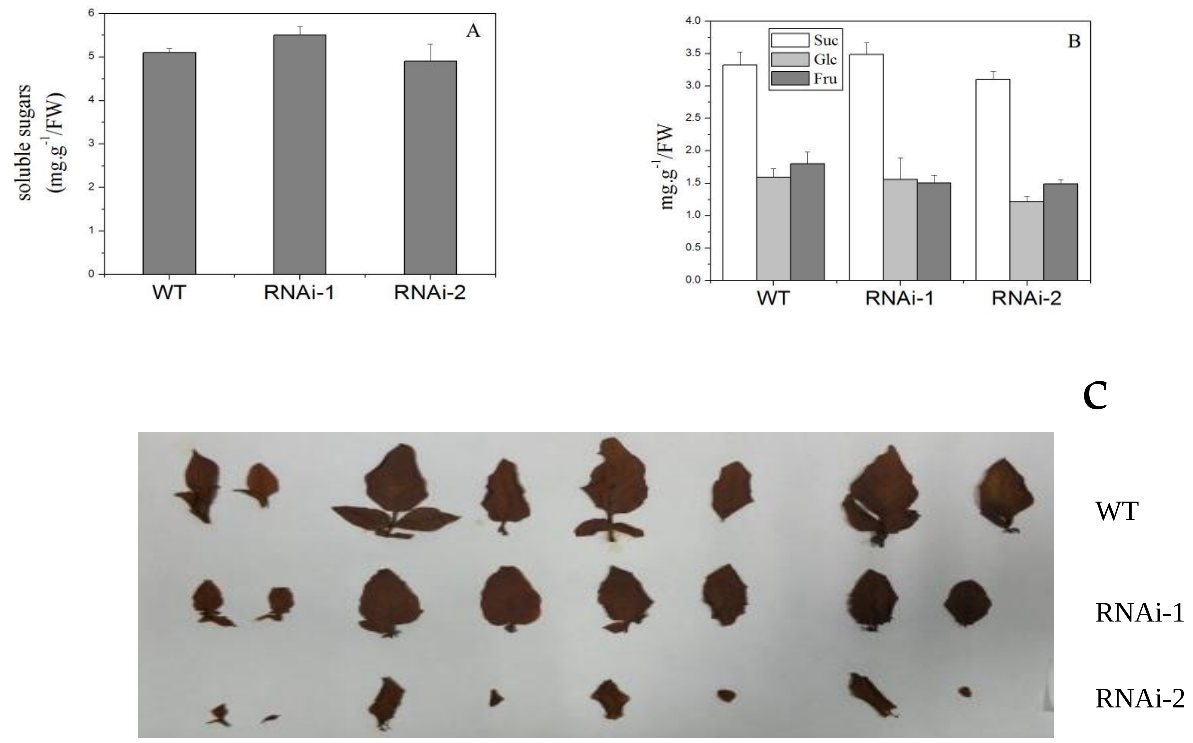

Figure S1. Carbohydrate content in leaves (A:Soluble sugar content; B:Sucrose, glucose and fructose content; C：Leaf starch staining).

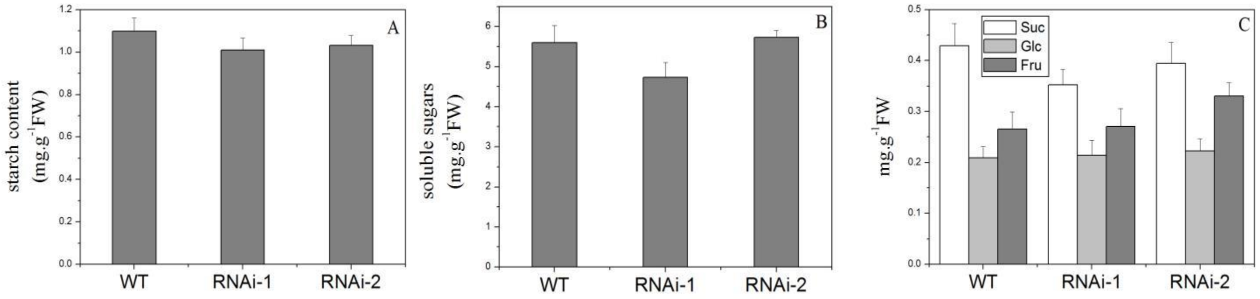

Figure S2. Carbohydrate content in tubers. (A:Starch content B:Soluble sugar content C:Sucrose, glucose and fructose content).
